# Two Ports Laparoscopic Inguinal Hernia Repair in Children

**DOI:** 10.1155/2015/821680

**Published:** 2015-02-16

**Authors:** Medhat M. Ibrahim

**Affiliations:** Pediatric Surgery Unit, Faculty of Medicine, Al-Azhar University, Nasr City, Cairo 11884, Egypt

## Abstract

*Introduction*. Several laparoscopic treatment techniques were designed for improving the outcome over the last decade. The various techniques differ in their approach to the inguinal internal ring, suturing and knotting techniques, number of ports used in the procedures, and mode of dissection of the hernia sac.* Patients and Surgical Technique*. 90 children were subjected to surgery and they undergone two-port laparoscopic repair of inguinal hernia in children. Technique feasibility in relation to other modalities of repair was the aim of this work. 90 children including 75 males and 15 females underwent surgery. Hernia in 55 cases was right-sided and in 15 left-sided. Two patients had recurrent hernia following open hernia repair. 70 (77.7%) cases were suffering unilateral hernia and 20 (22.2%) patients had bilateral hernia. Out of the 20 cases 5 cases were diagnosed by laparoscope (25%). The patients' median age was 18 months. The mean operative time for unilateral repairs was 15 to 20 minutes and bilateral was 21 to 30 minutes. There was no conversion. The complications were as follows: one case was recurrent right inguinal hernia and the second was stitch sinus.* Discussion*. The results confirm the safety and efficacy of two ports laparoscopic hernia repair in congenital inguinal hernia in relation to other modalities of treatment.

## 1. Introduction

During recent years, the trend toward laparoscopic approach for hernia repair in children has been increasingly justified. The ability to detect and repair the contralateral opening of internal rings simultaneously, along with safe high ligation of the hernia sac without injury of the vas deference or the spermatic vessels, make laparoscopic approach a reliable alternative to the conventional open techniques [1].

The universally known limitations of the laparoscopic surgery are as follows. (1) Most of these methods employ a laparoscope inserted via an umbilical incision and two lateral ports for instruments to ligate the hernia defect [2]. The necessity for intra-abdominal skills, such as intracorporeal suturing, knot tying, and manipulation of the suture on a needle may be time-consuming and cumbersome. (2) Recurrence rate after laparoscopic surgery is generally known to be higher than after open surgery [2].

With the increase in laparoscopic inguinal hernia repair, several treatment techniques have developed over the past two decades, aimed at improving the outcome [3]; the various techniques differ in their approach to the inguinal internal ring, suturing and knotting techniques, number of ports used in the procedures, endoscopic instruments used, mode of dissection of the hernia sac, and extracorporeal and intracorporeal suturing and knotting techniques [4].

This study is an early experience of two ports laparoscopic repair of inguinal hernia. I used two 5 mm ports which reduce the port numbers and size, the purse string suturing, and extracorporeal knotting of the hernia sac.

## 2. Materials and Surgical Technique

**Figure 1:**
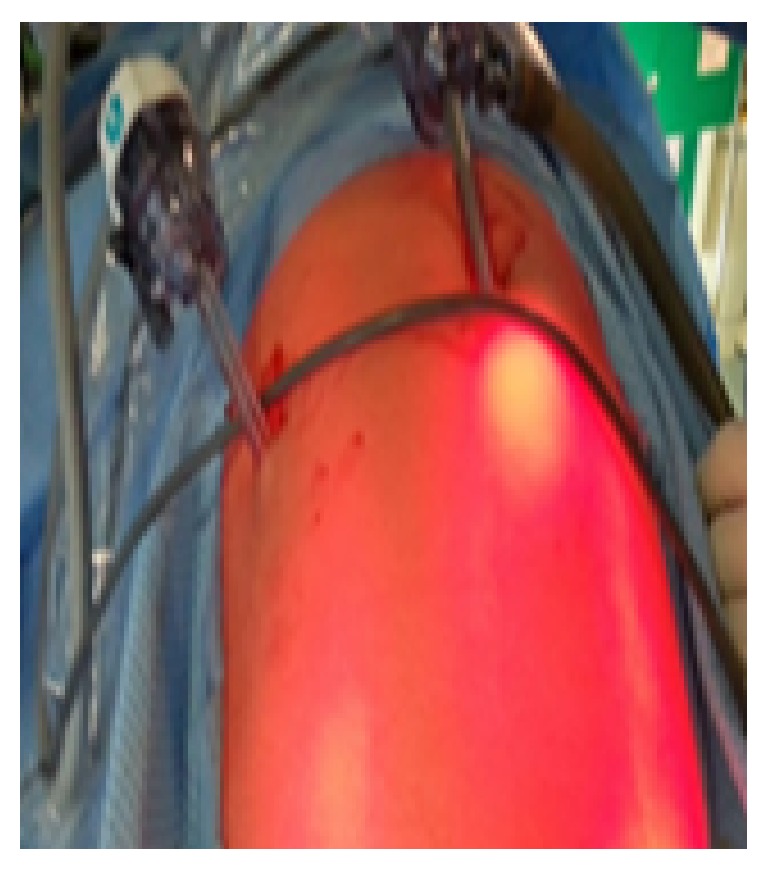
Port position used in right congenital inguinal hernia.

**Figure 2:**
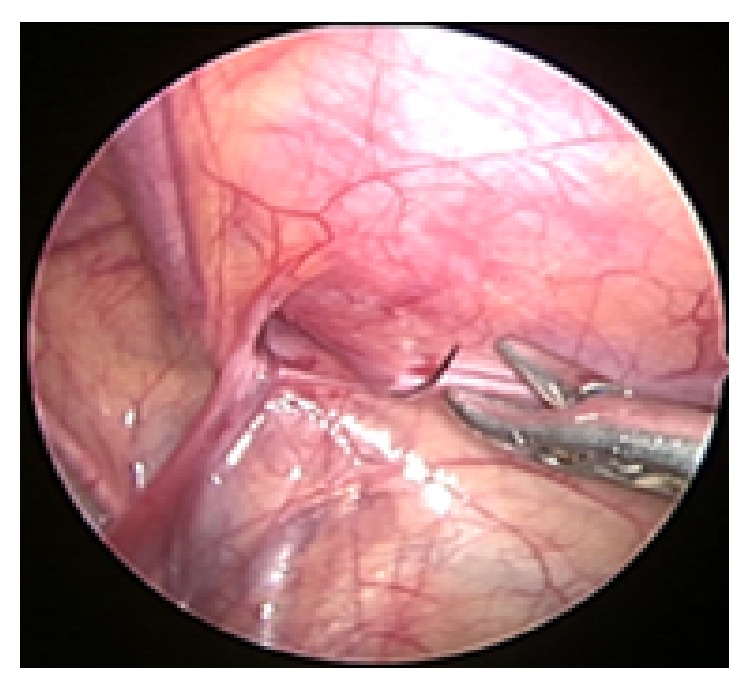
Needle passing to the peritoneal cavity.

This study was conducted from April 2009 to April 2013. 90 children with inguinal hernia were subjected to full clinical evaluation and routine investigations. The main outcome measurements were correlation between clinical diagnosis, abdominal ultrasound results and the laparoscopic evaluation of contralateral hernia, feasibility of two ports laparoscopic repair of the inguinal hernia, conversion rate, need for additional port, operative time, postoperative pain and requirement of analgesia, hospital stay, recurrence rate, and fat of nonexcised hernia sac and development of postoperative hydrocele. The ethical committee approved the technique. Written consent was obtained from the family after getting full information about the surgery and the postoperative squeal. All patients received one dose of antibiotic prophylaxis in the form of ceftriaxone 50 mg/kg at the time of induction of anesthesia. Under general endotracheal anesthesia, patients were scraped and draped. The surgeon position was at the child head. ENDOPATH XCEL port (5 mm) with 00 scope inserted supra umbilical by close technique. Pneumo-peritoneum pressure adjusted at 8–12 mm Hg according to child condition. Initial visualization of both internal rings was done for the bilateral hernia diagnosis. Seconded working port 5 mm was inserted according to the unilaterally and bilaterally hernia under visualization (Figure 1). Patient position was changed to trendelenburg position 45° which helped in reduction of the hernia contents and gave better visualization of the rings. Proline 3/0 suture mounted needle was passed from the outside through skin and abdominal wall muscles to the peritoneal cavity under visualization (Figure 2). Laparoscopic needle holder grasps the needle inside the abdominal cavity.

**Figure 3:**
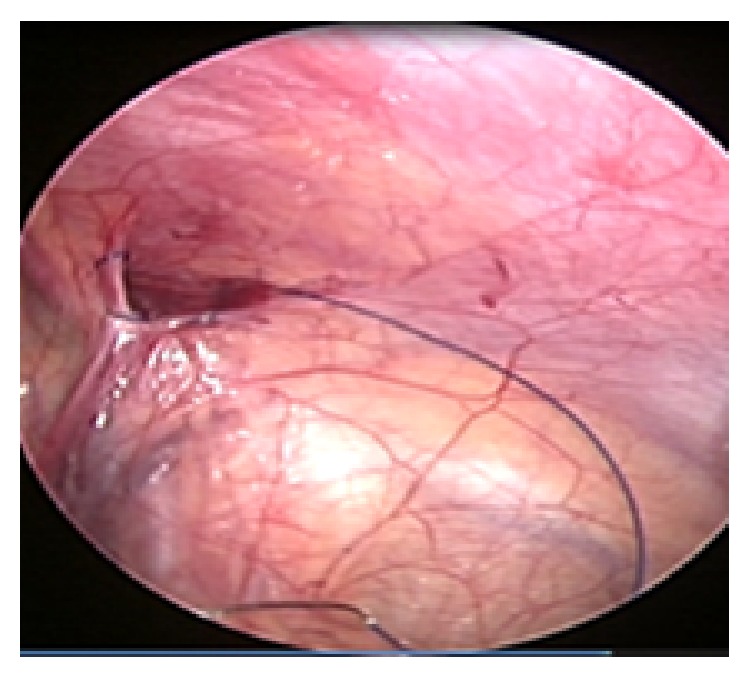
Proline purse string suture around the hernia neck.

**Figure 4:**
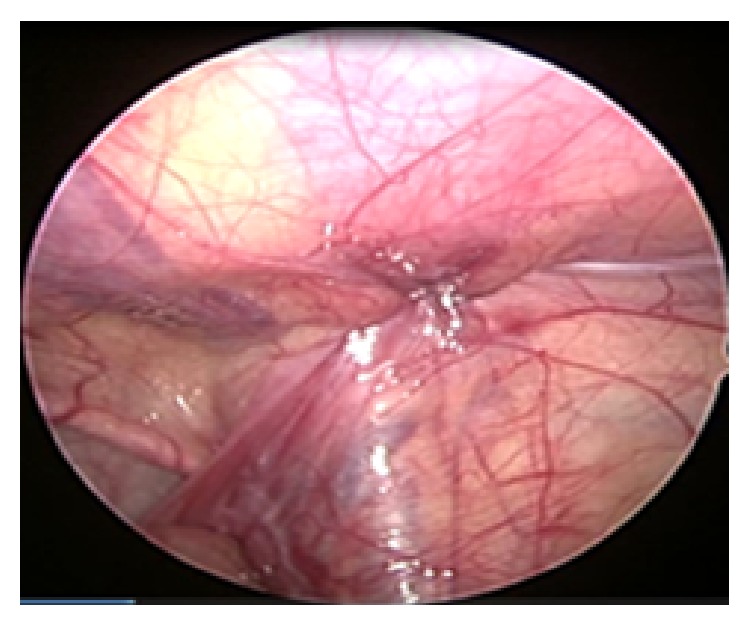
Closed hernia sac neck.

The hernial sac neck was closed by purse string 3/0 non- absorbable proline suture as high as possible (Figure 3). The suture was placed between the peritoneum and the fascia with preservation of the testicular vessels and the vas deference. The needle pushed out the abdomen by the laparoscopic needle holder until it appears outside the abdomen. Extracorporeal knot tying was done. The knot was fixed deep in the abdominal wall by small snip skin incision by number 11 knife. Full inspection of the abdominal wall and the closed defect was done (Figure 4). Laparoscopic abdominal exploration was done in all cases. All patients were asked to come for follow-up at outpatient clinic after 7 days, 2 weeks, 6 months, and 1 year. 90 children including 75 males and 15 females underwent two ports laparoscopic inguinal hernia repair. Hernia in 55 cases was right-sided and in 15 left-sided. Two patients had recurrence following previous hernia repair through groin incision. 70 (77.7%) cases were suffering unilateral inguinal hernia and 20 (22.2%) patients had bilateral inguinal hernia. Out of the 20 cases 5 cases were diagnosed by laparoscopic exploration (25%). These cases were diagnosed as left congenital inguinal hernia by clinical and ultrasound examinations while the laparoscope shows the metachronous hernia at the time of surgery.

The age of the patients ranged from 6 months to 8 years. The median age was 18 months. The mean operative time for unilateral repairs was (15 to 20 minutes) and bilateral hernia repair was (21 to 30 minutes). The scars on the abdominal wall were small and minute (5 mm incision for umbilical port, 5 mm working port and stitch site). There was no conversion or third port insertion. During follow-up there was one patient who got noncomplicated right recurrent inguinal hernia after two weeks from surgery. This patient underwent open right herniotomy where the hernia sac was not closed and proline stitch did not surround all the circumferences of the sac. Another case gets stitch sinuses at the proline knot site, which required open surgical removal of the stitch and open herniotomy at the same time. In this case the hernia sac was closed completely by the proline stitch. Once the proline stitch removed the sac opened, so, herniotomy and Trans fixation was done. There were no other complications such as testicular atrophy or secondary hydrocele. All patients were recovered smoothly from anesthesia and there was no need for analgesia. All patients were discharged home two hours after full recovery from anesthesia.

## 3. Discussion

Inguinal hernia in pediatric age group is a common problem and all the pediatric surgeons are fully familiar with the various aspects of its traditional surgical repair through the groin incision which has a high success rate and acceptable cosmetic results with few complications [4].

By far one of the drawbacks of this conventional technique is inability to rule out the contralateral patent processes vaginalis and synchronous hernia. With the advent of minimal access surgery, many pediatric surgeons accepted it, as a suitable and reliable alternative to the open techniques, considering its superiority for handling tissues during repair of recurrent inguinal hernias and also for its capabilities in regard to justifying and managing the synchronous subtle contralateral hernia [5]. However, there are still some issues regarding the introduction of laparoscopic inguinal hernia repair as the gold standard method, especially taking into consideration the possible longer operative time and the inevitable need for three separate ports which is the case in routine laparoscopic herniotomy techniques. In these works only two ports were used to do laparoscopic hernia repair in children, which increases the benefit of the laparoscopic herniotomy. The modified, new laparoscopic technique improved the diagnosis of ipsilateral hernia, using extracorporeal tying, that yields excellent cosmetic result.

Out of the 20 cases 5 (25%) case were diagnosed by laparoscopic exploration. These cases were diagnosed as left congenital inguinal hernia clinically and ultrasound examinations cannot detect the metachronous hernia in the right side prior to surgery. Laparoscope was superior to ultrasound in diagnosis of the metachronous hernia, while in other studies significant number of children (20%) presenting with unilateral hernias have contralateral patent processes vaginalis [5, 6]. The options for detection of contralateral patent processes vaginalis are many, namely, routine bilateral open surgery explorations [7], use of ultrasonography [8], laparoscopy [9], and the wait and watch policy [10].

Although laparoscopy proves advantageous over open surgery by precise detection and simultaneous repair of contralateral patent processes vaginalis, its management remains a contentious issue. The current consensus amongst surgeons practicing open surgery favors operating on the symptomatic side alone [9] as the rate of metachronous hernia is significant that it necessitates subsequent surgery in a twentieth of patients [11]. Therefore, this advantage of laparoscopic hernia repair may be significant in clinical practice as it gives good diagnosis and also repair for the metachronous hernia.

In open surgery, time is consumed in gaining access, obtaining adequate exposure, identification of the hernia sac, and dissection of the cord structures from the sac without harming the important cord structures [10]. In laparoscopic surgery, approaching from within makes the area of interest bloodless, and the magnification renders anatomy splendidly clear, making surgery precise [12]. But the time limiting step remains intracorporeal suturing that places considerable demands on the requirement of hand eye coordination, especially while negotiating the posterior and medial hemicircumference of the internal ring, over the iliac and inferior epigastric vessels [11]. With growing experience [11] and in this study the subfascia purse string suture and extracorporeal subcutaneous knotting with two ports markedly reduce the operative time over the traditional three ports laparoscopic hernia repair with intracorporeal suture ligation. In this study the mean operative time for unilateral repairs was (15 to 20 minutes) and bilateral hernia repair was (21 to 30 minutes); the mean duration of surgery was markedly less than the operative time noted in Lukong study [3]. The extracorporeal suturing was effective easy not need for special tool or surgical skills which also proved by other studys [11, 12].

The difference in postoperative pain following open surgery and laparoscopic surgery is subject to controversy. Some report less pain while others report greater pain in the immediate postoperative period following laparoscopic surgery compared with open surgery [13]. Bharathi R. found pain perception following either procedure to be similar. Parietal pain predominates in open surgery can well be controlled by caudal analgesia. On the other hand, pain perception is multimodal and multifactorial in laparoscopic surgery [14]. In addition to parietal pain caused by port placement, capnoperitoneum causes visceral pain due to stretching (peritoneal and diaphragmatic) and acidosis [14]. Neither the use of smaller ports nor the use of caudal analgesia would completely obliterate pain following laparoscopy [14]. Therefore, the decrease in the size of the incision does not necessarily translate into a proportionate decrease in pain. Hence, the difference in postoperative pain between laparoscopic surgery and open surgery is not significant enough to rate either surgery superior. In this study, the intraperitoneal gas pressure was located less than in other studies [14] due to the less number of ports and no need for intra-abdominal surgical maneuvers. Which reduced the post-operative pain. All patient was not need for post-operative analgesia.

Many new techniques have recently taken place in pediatric laparoscopic inguinal hernia surgery. Lee and Liang [15] introduced the Endo-needle designed specifically for laparoscopic extraperitoneal closure of the patent processus vaginalis. Endo and Ukiyama performed microlaparoscopic high ligation in 450 patients with good results. They reported no complications related to the procedure and a remarkably low recurrence rate (0.88%) [16]. Prasad et al. used a stainless steel curved awl and a 1.7 mm telescope to safely perform needlescopic inguinal herniorrhaphy [17]. Shalaby et al. used RN for closure of IIR in 150 patients successfully with excellent cosmetic results without any recurrence [18]. Chan and Tam [19] introduced the saline injection and needle sign to reduce the recurrence rate in his series. Bharathi et al. [20] used the TNH technique in 67 repairs, and 146 repairs were performed using the SEAL technique. They stated that SEAL resulted in marked reduction of operative time when compared to the TNH technique (unilateral: 15 versus 25 minutes; bilateral: 25 versus 40 minutes). They added that avoiding the vas deferens and gonadal vessels during the SEAL repair in boys may leave a small gap at the internal ring as well as leaving the hernia sac in situ, which has the potential to contribute to a higher incidence of recurrence in male patients. They believe that ligation of the internal ring leads to scarring and obliteration of the space distally. This would explain the relatively low incidence of postoperative hydrocele. Fluid accumulating in the distal sac postoperatively often reabsorbs spontaneously and does not necessitate additional intervention [20]. In this study there was no hydrocele detected as a postoperative complication in spite of no excision of the sac and the recurrence rate was 1% recurrence. Several piercings of the peritoneum by RN around the neck of the sac may add fixation of the suture at this level that prevents migration of the suture distally initiating recurrence. It may result in creation of adhesions of the sac preventing hydrocele formation. This may explain the high incidence of recurrence in the series of Manoharan et al. [9] and Bharathi et al. [20] that apply the subcutaneous suture around the internal ring without piercing the peritoneum. However, Chan and Tam [19] reported a very low recurrence rate (1%) after refinement of the technique by using TNH and injecting saline around the vas and vessels and using the needle sign to avoid damage to the testicular vessel and vas. They claimed that presence of a complete ring around IIR prevents recurrence. Prasad et al. reported no recurrence in their early small series of 8 cases [17]. Shalaby et al. reported no recurrence in their series [18]. The results of this study confirm the safety and efficacy of laparoscopic hernia repair with two ports in congenital inguinal hernia in children. It resulted in good diagnosis and management of metachronous hernia and marked reduction of operative time, less recurrence, no hydrocele formation, excellent cosmetic, and no postoperative pain results.
